# Constituents of *Xerolekia speciosissima* (L.) Anderb. (Inuleae), and Anti-Inflammatory Activity of 7,10-Diisobutyryloxy-8,9-epoxythymyl Isobutyrate

**DOI:** 10.3390/molecules25214913

**Published:** 2020-10-23

**Authors:** Natalia Kłeczek, Janusz Malarz, Barbara Gierlikowska, Anna K. Kiss, Anna Stojakowska

**Affiliations:** 1 Maj Institute of Pharmacology, Polish Academy of Sciences, Smętna Street 12, 31-343 Kraków, Poland; kleczek@if-pan.krakow.pl (N.K.); malarzj@if-pan.krakow.pl (J.M.); 2 Department of Pharmacognosy and Molecular Basis of Phytotherapy, Medical University of Warsaw, 1 Banacha Street, 02-097 Warsaw, Poland; bmichalak@wum.edu.pl (B.G.); akiss@wum.edu.pl (A.K.K.); 3 Department of Laboratory Diagnostics and Clinical Immunology of Developmental Age, Medical University of Warsaw, 63a Żwirki i Wigury Street, 02-091 Warsaw, Poland

**Keywords:** *Buphthalmum*, caffeic acid derivatives, flavonoids, Inuleae, leontopodic acid, sequiterpene lactones, *Telekia*, thymol derivatives

## Abstract

*Xerolekia speciosissima* (L.) Anderb., a rare plant from the north of Italy, is a member of the Inuleae-Inulinae subtribe of the Asteraceae. Despite its close taxonomic relationship with many species possessing medicinal properties, the chemical composition of the plant has remained unknown until now. A hydroalcoholic extract from the aerial parts of *X. speciosissima* was analyzed by HPLC-DAD-MS^n^, revealing the presence of caffeic acid derivatives and flavonoids. In all, 19 compounds, including commonly found chlorogenic acids and less frequently occurring butyryl and methylbutyryl conjugates of dicaffeoylquinic and tricaffeoylhexaric acids, plus two flavonoids, were tentatively identified. Chromatographic separation of a hydroalcoholic extract from the capitula of the plant led to the isolation of (+)-dehydrodiconiferyl alcohol 4-*O*-*β*-glucopyranoside, quercimeritrin, astragalin, isoquercitrin, 6-hydroxykaempferol-7-*O*-*β*-glucoside, quercetagitrin, methyl caffeate, caffeic acid, protocatechuic acid, chlorogenic acid and 1,5-dicaffeoylquinic acid. Composition of a nonpolar extract from the aerial parts of the plant was analyzed by chromatographic methods supported with ^1^H-NMR spectroscopy. The analysis revealed the presence of loliolide, reynosin, samtamarine, 2,3-dihydroaromaticin, 2-deoxy-4-*epi*-pulchellin and thymol derivatives as terpenoid constituents of the plant. One of the latter compounds—7,10-diisobutyryloxy-8,9-epoxythymyl isobutyrate—at concentrations 0.5, 1.0 and 2.5 μM, significantly reduced IL-8, IL-1β and CCL2 excretion by LPS-stimulated human neutrophils.

## 1. Introduction

*Xerolekia speciosissima* L. Anderb. (synonyms: *Buphthalmum speciosissimum* Ard., *Telekia speciosissima* (L.) Less.), a species which belongs to the newly created *Xerolekia* genus [1] is considered a member of the tribe Inuleae, subtribe Inulinae, of the family Compositae (Asteraceae) [2]. *X. speciosissima* is a perennial herb, 20–60 cm tall, with a stem having resin canals. Leaves of the plant are up to 25 cm long and are broadly lanceolate; inflorescences are solitary, terminal, heterogamous and have yellow flowers. The species, growing in nature, is a pre-Alpine endemite, listed in Italian and regional (Lombardy) red lists, that naturally inhabits crevices on limestone or dolomite rocks, between 1000 and 1900 m.a.s.l. Its distribution is limited to the area from Lake Como to Lake Garda and to the Ledro Valley [1,3].

*X. speciosissima* is a chemically yet unexplored plant closely related to numerous species rich in biologically active terpenoid and phenolic compounds of potential use in diverse fields, including the pharmaceutical and cosmetics industries. Some taxonomically connected genera, e.g., *Inula*, *Dittrichia* and *Carpesium*, are known from their medicinal uses in both traditional and conventional therapeutic systems of European and Far East countries [4,5]. Our previous investigation [6] on terpenoid metabolites from roots of *X. speciosissma* led to the isolation of five monoterpenoid thymol derivatives. Two of the compounds were formerly unknown from nature, and the remaining three were previously described as constituents of the Inuleae. Thymol derivatives have been frequently isolated from plants with known medicinal uses, e.g., *Inula* spp., *Carpesium* spp., *Eupatorium* spp. and *Arnica* spp. However, the literature data concerning their pharmacological activities are sparse [7].

The aims of the present study were to explore yet unknown constituents of the aerial parts and roots of the rare plant in a search for the active metabolites, and to assess the anti-inflammatory activity of the monoterpenoid thymol derivative synthesized by the plant.

## 2. Results

### 2.1. Phenolic Compounds in Roots and Aerial Parts of X. speciosissima

**Figure 1:**
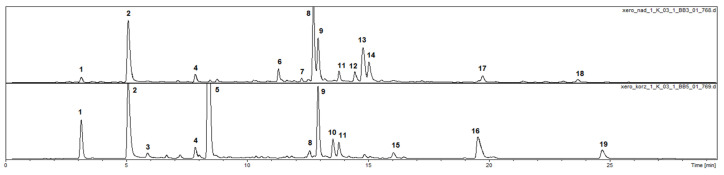
HPLC-DAD chromatogram of *Xerolekia speciocissima* extracts at a concentration of 10 mg/mL (2 μL injected) acquired at 325 nm: upper part—aerial parts; lower part—roots. Compounds: **1**—3-*O*-caffeoyl quinic acid, **2**—5-*O*-caffeoyl quinic acid, **3**—4-*O*-caffeoyl quinic acid, **4**—dicaffeoyl hexaric acid, **5**—1,3-di-*O*-caffeoyl quinic acid, **6**—quercetin hexoside, **7**—kaempferol rhamnosylhexoside, **8**—3,4-di-*O*-caffeoyl quinic acid, **9**—1,5-di-*O*-caffeoyl quinic acid, **10**—tricaffeoyl hexaric acid (I), **11**—4,5-di-*O*-caffeoyl quinic acid, **12**—dicaffeoyl quinic acid derivative, **13**—tricaffeoyl hexaric acid (II), **14**—tricaffeoyl hexaric acid (III), **15**—hydroxybutyryl-tricaffeoyl hexaric acid, **16**—tetracaffeoyl hexaric acid, **17**—isobutyryl-dicaffeoyl quinic acid, **18**—methylbutyryl/isovaleryl-dicaffeoyl quinic acid, **19**—isobutyryl-tricaffeoyl hexaric acid.

Total phenolic contents in leaves and roots of *X. speciosissima* were estimated as 61.66 ± 2.14 mg g^−1^ GAE and 80.08 ± 1.10 mg g^−1^ GAE, respectively. UHPLC-DAD-MS^n^ analysis of hydroalcoholic extracts from roots and aerial parts of the plant revealed the presence of 17 compounds with absorption maxima at 324–328 nm (caffeic acid derivatives). Twelve of the compounds were accumulated in the roots and eleven in the stems and leaves of the plant; six could be detected in both shoots and roots. Moreover, two compounds that demonstrated different UV-Vis spectral properties were observed (Figure 1, Table 1). The two compounds (peaks 6 and 7) that were present only in aerial parts of the plant, based on their UV spectra, quasimolecular ions at *m*/*z* 463 [M − H]^−^ and 593 [M − H]^−^, respectively, and product ions at *m*/*z* 301 and *m*/*z* 285, were identified as quercetin hexoside (peak 6) and kaempferol rhamnosylhexoside (peak 7). Peaks 1, 2 and 3 (*m*/*z* = 353 [M − H]^−^) were identified as signals of 3-*O*-caffeoylquinic acid (3-CQA; IUPAC numbering system), 5-*O*-caffeoylquinic acid (5-CQA) and 4-*O*-caffeoylquinic acid (4-CQA), respectively, whilst compounds 5, 8, 9 and 11 (*m*/*z* = 515 [M − H]^−^), taking into consideration fragmentation patterns of their quasimolecular ions (Table 1), proved to be four different isomers of di-*O*-caffeoyl quinic acid (DCQA)—namely: 1,3, 3,4, 1,5 and 4,5-di-*O*-caffeoylquinic acids [8,9]. Peaks 4, 10, 13, 14 and 16, representing compounds which showed cleavage of two, three or four caffeoyl [M − H − (2–4 × 162)]^−^ moieties resulting in *m*/*z* 209 fragments, were assigned to hexaric acid derivatives: di-*O* caffeoylhexaric acid (peak 4), tri-*O*-caffeoylhexaric acid (I) (peak 10), tri-*O*-caffeoylhexaric acid (II) (peak 13), tri-*O*-caffeoylhexaric acid (III) (peak 14) and tetra-*O*-caffeoylhexaric acid (peak 16). The compounds have been described earlier as constituents of other plants of the Asteraceae family, including systematically related species *Inula helenium* and *Carpesium divaricatum* [10,11,12,13]. The compound represented by peak 12 was an unknown derivative of dicaffeoylquinic acid judging from the presence of fragmentation ions at *m*/*z* 353, 335 and 179. Peak 15 was tentatively identified as a signal of hydroxybutyryl-tricaffeoyl hexaric acid (leontopodic acid) by looking at the fragmentation pattern of the compound described by Schwaiger et al. [14]. The compound corresponding to the peak 17 was tentatively identified as isobutyryl-dicaffeoylquinic acid based on the presence of quasimolecular ion at *m*/*z* 585 [M − H]^−^ and product ions at *m*/*z* 497 [585 − C_3_H_7_COOH]^−^, 423 [585 − caffeoyl]^−^, 335 [497 − caffeoyl]^−^, [423 − C_3_H_7_COOH]^−^, and 179 [caffeic acid − H]^−^, which is in accordance with the data given by Heilmann et al. [15]. The compound represented by peak 18 (*m*/*z* at 599 [M − H]^−^), which demonstrated a similar fragmentation pattern to that of 17, except for the fact that the product ion at *m*/*z* 497 was generated by the cleavage of 2-methylbutyryl or 3-methylbutyryl (isovaleryl) instead of isobutyryl moiety [599 − C_4_H_9_COOH]^−^, could be envisioned as 2-methylbutyryl/isovaleryl-di-O-caffeoylquinic acid. The compound represented by the signal 19 was probably deoxy derivative of leontopodic acid, judging from the *m*/*z* value of its quasimolecular ion (765 [M − H]^−^) and fragmentation ions at *m*/*z* 603 and 441 [13,14].

### 2.2. Caffeic Acid Derivatives in Leaves of X. speciosissima, Buphthalmum salicifolium and Telekia speciosa

Results of HPLC-DAD analysis of caffeic acid derivatives in young leaves of *X. speciosissima* and the two closely related species are summarized in Table 2. The plant material was collected in May, at the start of vegetation.

Moreover, five unidentified caffeic acid derivatives that were exclusively present in *T. speciosa* leaves and one specific for *B. salicifolium* were detected.

### 2.3. Constituents of a Hydroalcoholic Extract from Capitula of X. speciosissima

**Figure 2:**
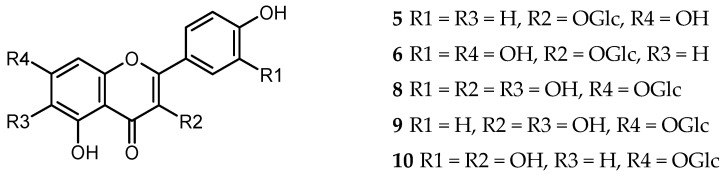
Chemical structures of flavonoids (**5**, **6**, **8**–**10**) isolated from capitula of *X. speciosissima*.

A hydroalcoholic extract from the dried inflorescences of *X. speciosissima* yielded caffeic acid and its derivatives, flavonols—derivatives of quercetin and kaempferol (see Figure 2), one lignan and one hydroxybenzoic acid. The isolated compounds were identified on the basis of their chromatographic behavior and their spectral data (UV, ^1^H-NMR 400.17 MHz), with reference to those of the standard compounds and to those from the literature. The only isolated lignan, of neolignan type, (+)-dehydrodiconiferyl alcohol 4-*O*-*β*-glucopyranoside (**1**) [16] and protocatechuic acid (3,4-dihydroxybenzoic acid, **2**) [17], were minor constituents of the examined extract. Caffeic acid (**3**) [18] and its methyl ester (**4**) [19] were present in majority of fractions eluted from the polyamide column. The most abundant conjugates of caffeic and quinic acids were 5-CQA (**7**) [20], and 1,5-DCQA (**11**) [21], which was the main component of the complex mixture of hydroxycinnamates from fractions P87–P91.

Three quercetin and two kaempferol derivatives were found in *X. speciosissima* capitula. Except for ubiquitous flavonol glucosides: isoquercitrin (quercetin 3-*O*-*β*-glucopyranoside, **6**) [17] and astragalin (kaempferol 3-*O*-*β*-glucopyranoside, **5**) [22], three less common compounds are worth mentioning: quercetagitrin (6-hydroxyquercetin 7-*O*-*β*-glucopyranoside, **8**), 6-hydroxykaempferol 7-*O*-*β*-glucopyranoside (**9**) [23] and quercimeritrin (quercetin 7-*O*-*β*-glucopyranoside, **10**) [24].

### 2.4. Constituents of a Chloroform Extract from Aerial Parts of X. speciosissima

**Figure 3:**
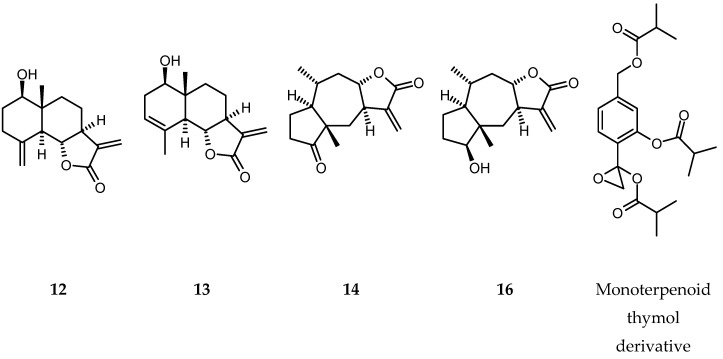
Chemical structures of sesquiterpene lactones (**12**–**14** and **16**) isolated from aerial parts of *X. speciosissima* and a monoterpenoid thymol derivative—7,10-diisobutyryloxy-8,9-epoxythymol isobutyrate.

Four sesquiterpene lactones: reynosin (**12**), santamarine (**13**) [25], 2,3-dihydroaromaticin (**14**) [26] and 2-deoxy-4-*epi*-pulchellin (**16**) [27] (see Figure 3) together with one apocarotenoide—loliolide (**15**) [28] were isolated from a chloroform extract of *X. speciosissima* aerial parts. The compounds were identified based on their experimental ^1^H-NMR spectroscopic data and their chromatographic parameters. The data were directly compared either with those of the standard compounds or with those from the literature. A pair of isomeric eudesmanolides, reynosin and santamarine, have been frequently found in different, taxonomically distant species, also outside the Asteraceae. It has been known for a long time that the compounds might be artifacts formed from costunolide-1,10-epoxide during chromatographic separation on silica gel [29]. This could explain their occurrence in fractions of different polarity. A mixture of monoterpenoid thymol derivatives was also separated from the extract. The mixture was not further purified, as it contained multiple compounds in relatively low amounts. Thymol derivatives were major terpenoid constituents in roots of *X. speciosissima* [6].

### 2.5. Effects of 7,10-Diisobutyryloxy-8,9-epoxythymyl Isobutyrate on Lipopolysaccharide (LPS)-Stimulated Release of Pro-Inflammatory Cytokines From Human Neutrophils

#### 2.5.1. Cytotoxicity

**Figure 4:**
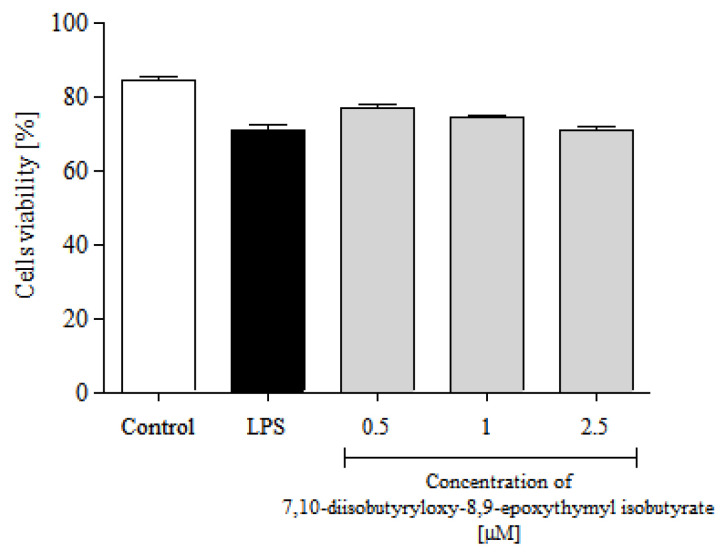
Cytotoxic effects of 7,10-diisobutyryloxy-8,9-epoxythymyl isobutyrate, at concentrations 0.5, 1.0 and 2.5 μM, on human LPS-stimulated neutrophils. Results shown as percentages of cells without diminished membrane integrity (propidium iodide negative cells). Control—untreated cells; LPS—cells stimulated with LPS (stimulated control). Statistical significance: * *p* < 0.05, with reference to a stimulated control.

The cytotoxicity of 7,10-diisobutyryloxy-8,9-epoxythymyl isobutyrate to human polymorphonuclear leukocytes (PMNs) was investigated using propidium iodide (PI) staining and flow cytometry (FACS) analysis. The compound did not cause toxicity in PMNs at 2.5 μM or lower concentrations (Figure 4). Solvent (DMSO) was not toxic to the cells either (data not shown). On the basis of these data, all further experiments were performed using the tested compound at concentrations up to 2.5 μM.

#### 2.5.2. Reactive Oxygen Species (ROS) Generation

**Figure 5:**
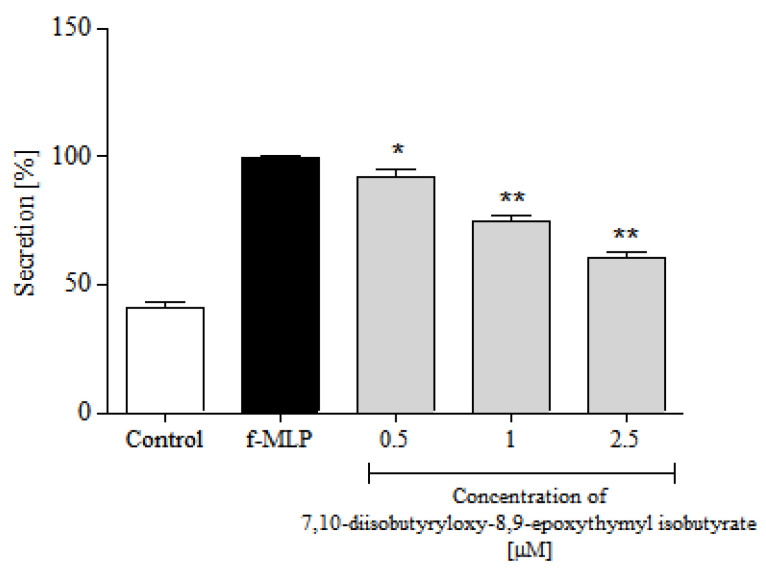
Inhibitory effects of 7,10-diisobutyryloxy-8,9-epoxythymyl isobutyrate, at concentrations 0.5, 1.0 and 2.5 μM, on the ROS release from f-MLP stimulated human neutrophils. Statistical significance: * *p* < 0.05, ** *p* < 0.001, with reference to a stimulated control.

Activation of PMNs at a site of inflammation induces an oxidative burst in these cells. The phenomenon is characterized by intense ROS generation and liberation of proteolytic enzymes from azurophilic granules. An effect of 7,10-diisobutyryloxy-8,9-epoxythymyl isobutyrate on ROS production in PMNs was assessed in response to *N*-formyl-Met-Leu-Phe (f-MLP) stimulation. The examined monoterpenoid efficiently reduced ROS release at 1–2.5 μM (Figure 5).

#### 2.5.3. Release of Selected Pro-Inflammatory Cytokines/Chemokines (IL-8, TNF-A, IL-1β, CCL2)

**Figure 6:**
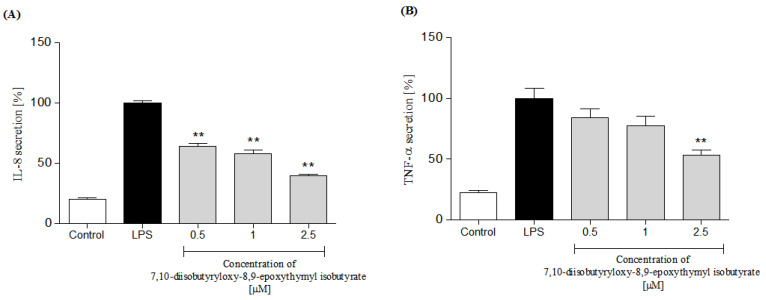
Inhibitory effects of 7,10-diisobutyryloxy-8,9-epoxythymyl isobutyrate, at concentrations 0.5, 1.0 and 2.5 μM, on IL-8 (**A**) and TNF-α; (**B**) secretion by LPS-stimulated human neutrophils. Statistical significance: ** *p* < 0.001, with reference to a stimulated control.

**Figure 7:**
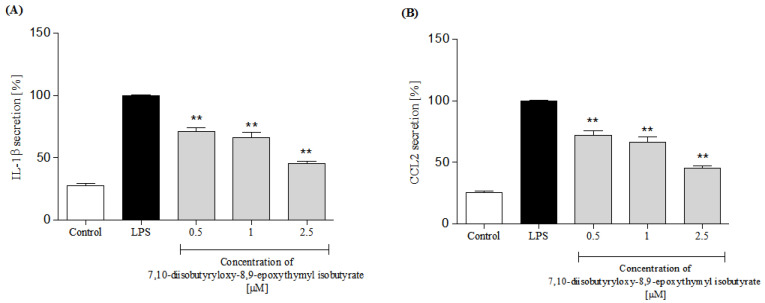
Inhibitory effects of 7,10-diisobutyryloxy-8,9-epoxythymyl isobutyrate, at concentrations 0.5, 1.0 and 2.5 μM, on IL-1β (**A**) and CCL2; (**B**) secretion by LPS-stimulated human neutrophils. Statistical significance: ** *p* < 0.001, with reference to a stimulated control.

In response to stimulation with pro-inflammatory agonists, e.g., LPS or f-MLP, human neutrophils secrete several cytokines and chemokines, including TNF-α, IL-1β, IL-8 and CCL2 [30,31]. In the present study, neutrophils were pretreated with the examined thymol derivative before their priming with LPS. Using ELISA, levels of IL-8, TNF-α, IL-1β and CCL2 were determined in the culture medium, 24 h after LPS stimulation. Preincubation of human neutrophils with 7,10-diisobutyryloxy-8,9-epoxythymyl isobutyrate resulted in significant and dose-dependent inhibition of IL-8 production upon LPS stimulation (Figure 6A). The compound was less active as an inhibitor of TNF-α production. Statistically significant reduction in release of this cytokine was achieved only with the highest of the tested concentrations (2.5 μM, see Figure 6B). Both IL-1β and CCL2 production by LPS treated neutrophils were significantly and dose-dependently lowered by pretreatment with the examined monoterpenoid at a dose of 0.5–2.5 μM (Figure 7A,B).

## 3. Discussion

Total phenolic contents in shoots and roots of *X. speciosissima* were higher than those estimated for *Cichorium intybus*, a food plant rich in polyphenols (approximate total phenolic content (TPC) value: 7.5–42.0 mg g^−1^ GAE) [32] and the other Asteraceae species studied, in which TPC values ranged from 3.7 to 15.2 GAE (mg g^−1^) [33,34]. In contrast to chicory plants, roots of *X. speciosissima* demonstrated higher reducing capacity (80.08 ± 1.10 mg g^−1^ GAE) than the aerial parts of the plant (61.66 ± 2.14 mg g^−1^ GAE).

As can be seen in the Table 2, caffeic acid derivatives constitute major part of the polyphenolic fraction from the plant shoots. By looking at the hydroxycinnamate profiles of *X. speciosissima*, *T. speciosa* and *B. salicifolium,* one can observe that *X. speciosissima* shares more similarity with *B. salicifolium*. Isobutyryl- and 2-methylbutyryl/isovaleryl-dicaffeoylquinic acids especially seem to be distinctive metabolites of the two species. The compounds were also found in *Carpesium divaricatum* Sieb. and Zucc. [13], another closely related species of the Inuleae-Inulinae. On the other hand, acylated glycosides of caffeic and ferulic acids [35] appear to be characteristic metabolites of *T. speciosa*.

Except for hydroxycinnamates, HPLC-DAD-MS^n^ analysis revealed the presence of two flavonol glycosides, in aerial parts of *X. speciosissima*. Flavonols, namely, 3-*O*-*β*-glucopyranosides of quercetin and kaempferol and 7-*O*-*β*-glucopyranosides of quercetin, quercetagetin (6-hydroxyquercetin) and 6-hydroxykaempferol, were also found in capitula of the plant. In contrast to *B. salicifolium*, flowerheads of *X. speciosissima* did not accumulate acylated flavonol glucosides [15]. Moreover, methylated flavonols (patuletin, isorhamnetin) were not found in the investigated plant material. Chemical diversity of the flavonoids produced by *X. speciosissima* is limited also in comparison with that of *T. speciosa* [36].

The majority of phenolic constituents found in *X. speciosissima* possess well documented biological activity, especially as antioxidative, free radical scavenging and anti-inflammatory agents [24,37,38,39,40]. Astragalin, isoquercitrin, protocatechuic acid and caffeoylquinic acids are among the most extensively studied plant metabolites, with respect to the potential health implications of their dietary intake.

Sesquiterpene lactones are a group of terpenoid metabolites considered to be useful taxonomic markers within the Asteraceae. In *X. speciosissima* the compounds are represented by two eudesmanolides and two pseudoguaianolides. The lactones were isolated exclusively from aerial parts of the plant. In the subaerial organs, monoterpenoid thymol derivatives were the only lower terpenoids that we managed to isolate [6]. Reynosin and santamarine, the two eudesmanolides found in stems and leaves of *X. speciosissima*, might have been partly of artifactual origin. Their occurrence in apolar fractions of the plant extract may suggest the presence of their germacranolide precursor costunolide-1,10-epoxide in the analyzed material. Both reynosin and santamarine, as was mentioned before, are of limited usefulness as taxonomic markers due to their occurrence in a number of taxonomically distant species. The compounds demonstrated anti-inflammatory activity in in vitro assays [25,41]. Reynosin inhibited platelet aggregation induced by arachidonic acid, ADP and platelet activating factor (PAF) [42], and protected neurons against dopamine-induced toxicity [43]. The two remaining lactones, pseudoguaianolides, 2,3-dihydroaromaticin and 2-deoxy-4-epi-pulchellin, are frequently found in plants of Inulae-Inulinae, e.g., in *Carpesium* spp., *Inula* spp., *Ondetia linearis* Benth and *T. speciosa* [44,45,46,47,48,49,50]. The compounds were active in in vitro assays as antiproliferative and anti-inflammatory agents [45,46,47,48].

Phytochemical analysis of aerial parts and flowerheads of *B. salicifolium* did not reveal the presence of sesquiterpene lactones [15,51]. Four bithiophenes were major non-polar constituents of the plant shoots. In contrast to *B. salicifolium*, *T. speciosa* is rich in sesquiterpene lactones of eudesmanolide, pseudoguaianolide and xanthanolide types [39,52]. The compounds could be found in both aerial and subaerial parts of the plant. The roots of *T. speciosa* contain a large amount of isoalantolactone and may be considered a substitute for *Inula helenium* roots. *X. speciosissima* is rather a poor source of sesquiterpene lactones when compared to *T. speciosa* and *Carpesium* spp.

Monoterpenoid thymol derivatives are synthesized in *B. salicifolium* [15], *T. speciosa* [53], *Carpesium* spp., [5] *Inula* spp. [4], *X. speciosissima* [6] and many other species. Outside the Inuleae, the compounds are frequently found in the Eupatorieae, Helenieae and other members of the Heliantheae alliance. Despite the frequent occurrence in many species of known medicinal use, only few studies have been devoted to the biological activities of the compounds [7]. Recently, a thymol derivative, 8-hydroxy-9,10-diisobutyryloxythymol (constituent of *X. speciosissima*), was found to inhibit the interaction between p53 (tumor suppressor protein) and its inhibitor, MDM2 protein [54]. Our investigation on anti-inflammatory activity of 7,10-diisobutyryloxy-8,9-epoxythymyl isobutyrate was meant to support the role of monoterpenoid thymol derivatives as active ingredients of plant preparations.

Polymorphonuclear leukocytes (PMNs) play a pivotal role in human immune system. They, among others, participate in fine regulation of the immune response and inflammatory process via the capability to respond to and to produce a variety of cytokins [31]. Cytokines produced by human neutrophils include pro-inflammatory cytokines IL-1β and TNF-α, and chemokines CCL2 and IL-8 (CXCL8) that are implicated in the pathogenesis of inflammatory diseases in humans. To assess an effect of 7,10-diisobutyryloxy-8,9-epoxythymyl isobutyrate on neutrophile function, secretion of the mentioned cytokines by LPS-stimulated human neutrophils in the absence or presence of the tested compound was monitored. Preincubation of human neutrophils with the examined thymol derivative significantly, and in a concentration-dependent manner, diminished f-MLP-induced ROS production by the cells (Figure 5). The tested compound significantly reduced secretion of pro-inflammatory cytokine IL-1β and chemokines CCL2 and IL-8 (Figure 6A and Figure 7). Only its highest concentration (2.5 μM) significantly affected secretion of TNF-α by LPS-stimulated neutrophils (Figure 6B). This might be due to very small amounts of TNF-α produced by human neutrophils. The results of our tests suggested that monoterpenoid thymol derivatives, together with sesquiterpene lactones and phenolic compounds, are involved in the anti-inflammatory activity of the Inuleae.

## 4. Materials and Methods

### 4.1. General Methods

NMR spectra were recorded in either CDCl_3_ or MeOD, on a Bruker AVANCE III HD 400 (Bruker Corp., Billerica, MA, USA), at resonance frequency of 400.17 MHz for ^1^H. Optical rotation was determined in MeOH on a PolAAr31 polarimeter (Optical Activity Ltd., England). RP-HPLC separations were performed using an Agilent 1200 Series HPLC system (Agilent Technologies, Santa Clara, CA, USA) equipped with a diode array detector. Analytical chromatographic separations were carried out on either a Kinetex XB-C18 column (4.6 × 250 mm, 5 μm total particle size; Phenomenex Inc., Torrance, CA, USA; nonpolar compounds) or a Zorbax Eclipse XDB-C18 column (4.6 × 150 mm; Agilent Technologies, Santa Clara, CA, USA; phenolic compounds). Semipreparative RP-HPLC was conducted on a Synergi 4μ Fusion-RP, 80 A, 250 × 10 mm column (Phenomenex Inc., Torrance, CA, USA), with an isocratic elution, using MeOH-H_2_O mixtures of different polarities. Conventional column chromatography (CC) was carried out on Silica gel 60 (0.063–0.2 mm, Merck, Germany), Polyamide 6 (Sigma-Aldrich Co., Saint Louis, MO, USA) and Sephadex LH-20 (GE Healthcare, Uppsala, Sweden). TLC separations were performed using precoated plates (Silica gel 60 without fluorescence indicator, Art. No 5553; Merck, Darmstadt, Germany).

### 4.2. Chemicals and Solvents

Chlorogenic acid (5-CQA, purity > 97% by HPLC), caffeic acid (purity ≥ 95%) and 1,3-DCQA (cynarin, purity > 99% by HPLC) were purchased from Roth (Karlsruhe, Germany). Folin–Ciocalteu reagent was supplied by Sigma-Aldrich Co. (St. Louis, MO, USA). MeOH of analytical grade was purchased from Avantor Performance Materials S.A. (Gliwice, Poland). Water was purified by a Mili-Q system (Milipore Corp., Bedford, MA, USA). MeOH and MeCN of HPLC grade and formic acid and glacial acetic acid were bought from Merck (Darmstadt, Germany). 7,10-Diisobutyryloxy-8,9-epoxythymyl isobutyrate (purity > 90% by HPLC) was isolated in our laboratory from roots of *X. speciosissima*. Phosphate-buffered saline (PBS) was bought from Biomed (Lublin, Poland). Hanks’ balanced salt solution (HBSS), RPMI 1640 medium, f-MLP (formyl-Met-Leu-Phenylalanine), LPS (from *Escherichia coli* 0111:B4), propidium iodide (PI), luminol, 4-(2-hydroxyethyl)-1-piperazineethanesulfonic acid (HEPES) solution and L-glutamine were purchased from Sigma-Aldrich Co. (St. Louis, MO, USA). Fetal bovine serum (FBS) was delivered by Gibco (Grand Island, NY, USA).

### 4.3. Plant Material

Seeds of *X. speciosissima* were supplied by the Alpine Botanical Garden “Rezia” (Bormio, Italy). That garden specializes in preservation of the plant species occurring in the Stelvio National Park (Lombardy, Italy). *X. speciosissima* seeds were germinated following the protocol by Brusa et al. [3]. The seedlings were initially grown in a glasshouse, and later on were planted into the garden. Plants for the phytochemical analysis were collected in the second season of vegetation, in June 2016, from the Garden of Medicinal Plants, Maj Institute of Pharmacology, Polish Academy of Sciences, Kraków, where the voucher specimen (1/16) was deposited. Roots; stems with leaves and buds; and capitula in bloom were separated and dried under shade, at room temperature.

For the HPLC-DAD analysis of caffeic acid derivatives in leaves of *X. speciosissima,* and in leaves of *Telekia speciosa* (Schreb.) Baumg. and *Buphthalmum salicifolium* L., two species closely related to the examined taxon [2], plant material was collected in May 2017 from perennial plants cultivated in the Garden of Medicinal Plants, Institute of Pharmacology, Polish Academy of Sciences, Kraków. Samples of the leaves were harvested from five individual plants of each of the investigated species at the rosette stage. Each sample (4 or 5 tiny leaves collected from one plant) was processed separately. Results are means of five measurements (± SD).

### 4.4. Estimation of Total Phenolic Content (TPC)

The reducing capacity of the plant material, referred as TPC, was estimated by using a Folin–Ciocalteu colorimetric method. The dry plant material (0.01 g) was extracted twice, for 2 h, with 2 mL of 80% MeOH containing 1% HCl, at room temperature, on a reciprocal shaker. The combined extracts were further analyzed as described by Velioglu et al. [55]. Results are expressed as mg of gallic acid equivalents (GAE) per 1 g of the plant material dry weight and are means of three measurements (± SD).

### 4.5. Phenolic Compounds Analysis by HPLC

#### 4.5.1. Preparation of Samples for HPLC-DAD and UHPLC-DAD-MS^n^ Analysis

The dry and pulverized plant material (0.1 g) was extracted twice with 10 mL of 70% MeOH, at room temperature, for 3 h, on a rotary shaker (100 r.p.m.). The extracts were combined and evaporated to dryness under reduced pressure, to give a residue which was either redissolved in 1 mL of 70% MeOH and centrifuged (11,340× *g*, 5 min) prior to analytical HPLC/DAD separation, or had an aliquot (0.01 g) dissolved in a mixture of MeOH and 0.1% HCOOH (8:2), filtered through 0.45 μm Chromafil membrane (Machery-Nagel, Duren, Germany) and subjected to UHPLC-DAD-MS^n^ analysis.

#### 4.5.2. Characterization of *X. speciosissima* Shoot and Root Extracts by UHPLC-DAD-MS^n^ Method

UHPLC-DAD-MS^n^ analysis was performed on UHPLC-3000 RS system (Dionex, Germany) with DAD detection and an AmaZon SL ion trap mass spectrometer with ESI interface (Bruker Daltonik GmbH, Germany). Separation was performed on a Kinetex XBC18 column (150 × 2.1 mm, 1.7 μm) Phenomenex (Torrance, CA, USA). The column temperature was 25 °C. The mobile phase (A) was H_2_O/HCOOH (100:0.1, *v*/*v*) and the mobile phase (B) was MeCN/HCOOH (100:0.1, *v*/*v*). A gradient system was used: 0–10 min 10–25% B; 10–40 min 25–35% B. The flow rate was 0.3 mL min^−1^. The column was equilibrated for 7 min between injections. UV spectra were recorded over a range of 200–450 nm; chromatograms were acquired at 325 nm. The LC eluate was introduced directly into the ESI interface without splitting. The nebulizer pressure was 40 psi; dry gas flow 9 L min^−1^; dry temperature 300 °C; and capillary voltage 4.5 kV. Analysis was carried out using scan from *m*/*z* 90 to 2,200. Compounds were analyzed in negative ion mode. The MS^2^ fragmentation was obtained for the most abundant ion at the time.

#### 4.5.3. Qualitative Analysis of *X. speciosissima*, *T. speciosa* and *B. salicifolium* Leaf Extracts and Quantification of Commonly Distributed Caffeic Acid Derivatives by HPLC-DAD

HPLC-DAD separations of the samples were performed using an Agilent 1200 Series HPLC instrument (Agilent Technologies, USA) equipped with a Rheodyne manual sample injector, column oven and a diode array detector. Quantitative analyses were carried out at 25 °C, on a Zorbax Eclipse XDB-C18 column, with a mobile phase consisting of H_2_O/HCOOH/CH_3_COOH 99/0.9/0.1 (solvent A) and MeCN/MeOH/HCOOH/CH_3_COOH 89/10/0.9/0.1 (solvent B), at a flow rate of 1 mL min^−1^, using 5 µL injections. The gradient elution conditions described by Spitaler et al. [56] were applied. The compounds were identified based on their retention time values, online UV spectra, co-chromatography with standard samples and comparison with the results of HPLC-DAD-MS^n^ analysis. Quantification was done using an external standard method as it was described previously [12].

### 4.6. Isolation of Chemical Constituents from a Hydroalcoholic Extract of X. speciosissima Inflorescences

Dried capitula of *X. speciosissima* (65 g) were powdered and exhaustively extracted at room temperature, with shaking, first with CHCl_3_ and subsequently with 70% (*v*/*v*) MeOH. The chloroform extract, containing mainly triterpenoid alcohols and their esters with fatty acids, was not further analyzed. The hydroalcoholic extract was evaporated in vacuo to provide 15 g of an oily residue. The residue was initially fractionated by CC on Polyamide using MeOH-H_2_O mixtures of decreasing polarity (0–90% MeOH). Collected fractions, P1–P100 (100 mL each), were combined according to their HPLC–DAD profiles and further separated by CC on Sephadex LH-20. Fractions P8–P12 (0.20 g; eluted with H_2_O), after CC on Sephadex with water as an eluent, gave **1** (3.1 mg). Fractions P37–P38 (0.14 g), obtained by elution with 50% MeOH (*v*/*v*) and subsequently purified by gel filtration, yielded: **2** (3.1 mg), a mixture containing **3** as a main constituent (8.0 mg), and **4** (6.8 mg). From fractions P65–P66 (0.15 g), eluted with 75% MeOH (*v*/*v*), after further separation, additional amounts of **3** (20.7 mg) and **4** (8.0 mg) together with flavonoids: a mixture of **5** and **6** (c. 3:1, 9.8 mg) and pure **6** (17.6 mg) were obtained. Fractions P67–P73 (0.12 g), subfractionated on Sephadex, furnished: **7** (10.0 mg), more of **6** (20.1 mg), a mixture (3.8 mg) of **8** and **9** (c. 1.6:1) and a fraction (3.2 mg) containing **10**. Fractions P87–P91 (0.14 g; eluted with MeOH), after further separation, yielded a mixture of hydroxycinnamates (34.3 mg) with **11** as a major constituent.

### 4.7. Isolation of Chemical Constituents from a Chloroform Extract of X. speciosissima Aerial Parts

Dried and pulverized aerial parts (325 g) of *X. speciosissima* (without capitula in bloom) were extracted five times with 1.7 L of CHCl_3_ at room temperature with shaking. The combined extracts were concentrated in vacuo, at 40 °C, providing c. 35 g of an oily residue. The residue was subjected to CC on silica using gradients of EtOAc in *n*-hexane (up to 100% EtOAc) and subsequently MeOH in EtOAc (up to 10% of MeOH). The separated fractions (50 mL each) were combined, as shown by TLC, and further fractionated either by preparative TLC or by semipreparative RP-HPLC. Fractions 74–79 and 86–87, eluted with *n*-hexane-EtOAc 9:1 (*v*/*v*) and *n*-hexane-EtOAc 4:1 (*v*/*v*), respectively, were subjected to prep TLC (*n*-hexane-EtOAc, 4:1) to give subfractions containing monoterpene thymol derivatives that were previously isolated from roots of the plant [6]. The subfractions were not further separated. Fractions 104–107 (0.42 g), eluted with *n*-hexane-EtOAc, 4:1 (*v*/*v*), were first subjected to preparative TLC (*n*-hexane-EtOAc, 4:1) and then to semipreparative HPLC (MeOH-H_2_O 7:3, flow rate 2 mL min^−1^) to yield **12** (2.0 mg) and **13** (2.7 mg). Fractions 114–117 (0.22 g), eluted with *n*-hexane-EtOAc 7:3 (*v*/*v*), were processed the same way as it was described above to give an additional amount of **12** (1 mg). Further elution of the column with *n*-hexane-EtOAc 3:2 (*v*/*v*) (fractions 141–144, 0.03 g), after purification by semipreparative HPLC (MeOH-H_2_O 7:3 (*v*/*v*), flow rate 2 mL min^−1^), allowed isolation of **14** (1.7 mg). Fractions 172–184 (0.09 g), eluted with *n*-hexane-EtOAc 1:1 (*v*/*v*), after further separation by semipreparative HPLC (MeOH-H_2_O 3:2 (*v*/*v*), flow rate 2 mL min^−1^) furnished **15** (10.7 mg), **16** (4.6 mg) and **12** (3.8 mg).

### 4.8. Assessment of the Effects of 7,10-Diisobutyryloxy-8,9-epoxythymyl Isobutyrate on Lipopolysaccharide (LPS)-Stimulated Release of Pro-Inflammatory Cytokines from Human Neutrophils

#### 4.8.1. Isolation of Human Neutrophils

Peripheral venous blood was obtained from healthy human donors (18–35 years old) in the Warsaw Blood Donation Centre. Donors did not smoke or take any medications. They were clinically recognized to be healthy and a routine laboratory tests showed all values to be within the normal ranges. Neutrophils were isolated by dextran sedimentation and centrifugation in a Ficoll Hypaque gradient and then resuspended in (Ca^2+^)-free HBSS buffer or RPMI 1640 medium. Blood samples from three donors were used in each experiment.

#### 4.8.2. Cytotoxicity Measurement

Cytotoxicity was assessed by a standard flow cytometric probe using propidium iodide (PI) staining. After 24 h of incubation in the absence or presence of the tested compound (at concentrations of 0.5, 1.0 and 2.5 μM), the neutrophils (3.5 × 10^5^) were harvested and centrifuged (1500 r.p.m.; 10 min; 4 °C), washed once with cold PBS and resuspended in 400 μL of PBS. A 5 μL aliquot of PI solution (50 μg/mL) was added to the cell suspension. After 15 min of incubation, in the dark, with PI at room temperature, cells were analyzed by BD FACSCalibur flow cytometer (BD Biosciences, San Jose, CA, USA) and 10,000 events were recorded per sample. The number of cells that displayed high permeability to PI, expressed as a percentage of PI(+) cells, was determined.

#### 4.8.3. ROS Production by Neutrophils

ROS production was measured using luminol-dependent chemiluminescence test. A 70 μL aliquot of neutrophil suspension (3.5 × 10^5^) in (Ca^2+^)-free HBSS buffer, 50 μL of the tested compound solution and 50 μL of luminol (100 μM) were added to a well in a 96 well plate. ROS production was initiated by the addition of f-MLP (30 μL of 0.1 μg/mL solution) to obtain a total volume of 200 μL per well. Chemiluminescence changes were measured for 40 min, at 2 min intervals, in a microplate reader (37 °C). The background chemiluminescence produced by non-stimulated cells was also determined. The percentage of inhibition was calculated by comparison to the stimulated control without the tested compound, at the maximum luminescence.

#### 4.8.4. IL-8, IL-1β, CCL-2 and TNFα Production by Neutrophils

Neutrophils (2 × 10^6^) were cultured in 24-well plates in RPMI 1640 medium with 10% FBS, 10 mM HEPES, and 2 mM L-glutamine, in the presence or absence of LPS (100 ng/mL) and in the absence or presence of 7,10-diisobutyryloxy-8,9-epoxythymyl isobutyrate (final concentration in a range of 0.5–2.5 μM), at 37 °C with 5% CO_2_. After 24 h, the supernatants were harvested and centrifuged (2000 r.p.m.; 10 min; 4 °C). The amounts of released cytokines were measured by enzyme-linked immunosorbent assay (ELISA) following the manufacturer’s instructions (BD Biosciences, USA). The effects on IL-8, IL-1β, CCL2 and TNF-α production were calculated by comparing the percentages of the released agents to the stimulated control, which lacked the test compound.

#### 4.8.5. Statistical Analysis

The results were expressed as the mean ± SEM of three independent experiments performed at least in duplicate. All analyses were performed using Statistica 13 software. The statistical significance of the differences between means was established by ANOVA with Dunnett’s post hoc test *p* values.

## 5. Conclusions

Our research supported a close relationship of *X. speciosissima* with *Carpesium* spp. and some species of the *Inula* genus. The species clearly differs from *B. salicifolium* with respect to sesquiterpene lactone and tiophene content. In contrast to *T. speciosa* (and resiniferous species of *Inula*), *X. speciosissima* does not accumulate substantial amounts of essential oil, rich in eudesmanolides, in its roots. The composition of polyphenolic fraction of *X. speciosissima*, despite some differences, places the taxon close to *Carpesium* spp. and *B. salicifolium.* The results of the phytochemical investigation are in agreement with the current taxonomic position of *X. speciosissima* as a separate monotypic genus.

*X. speciosissima*, a rare plant of the pre-Alpine area, produces specialized metabolites typical of the Inuleae. The majority of them, obtained from other sources, have been extensively studied in respect of their pharmacological activity in vitro. The most distinctive chemical feature of the plant is the occurrence of thymol derivatives of uncommon structures in its roots.

## Figures and Tables

**Table 1:** Retention times, UV maxima and MS^n^ data, in the negative ion mode, for the phenolic compounds present in extracts from *Xerolekia speciosissima* roots and aerial parts.

No	Compound	t*_R_* [min]	UV [nm]	[M − H]^−^	Product Ion Main Peaks ^1^	R ^2^	A ^3^
1	3-*O*-Caffeoylquinic acid (3-CQA)	3.1	325	353	**191**, 179	+	+
2	5-*O*-Caffeoylquinic acid (5-CQA)	5.1	325	353	**191**	+	+
3	4-*O*-Caffeoyl quinic acid (4-CQA)	5.9	325	353	191, 179, **173**	+	−
4	Dicaffeoyl hexaric acid	7.9	324	533	**371**, 209	+	+
5	1,3-Di-*O*-caffeoyl quinic acid (1,3-DCQA)	8.4	322	515	**353**, 335, 191, 179	+	−
6	Quercetin hexoside	11.3	274, 343	463	**301**	−	+
7	Kaempferol rhamnosylhexoside	12.3	265, 341	593	**285**	−	+
8	3,4-Di-*O*-caffeoyl quinic acid (3,4-DCQA)	12.5	325	515	**353**, 335, 299, 255, 203, 191, 179, 173	+	+
9	1,5-Di-*O*-caffeoyl quinic acid (1,5-DCQA)	12.9	327	515	**353**, 191	+	+
10	Tricaffeoyl hexaric acid (I)	13.5	327	695	**533, 371**, 209	+	−
11	4,5-Di-*O*-caffeoyl quinic acid (4,5-DCQA)	13.8	327	515	**353**, 317, 299, 255, 203, 173	+	+
12	Dicaffeoyl quinic acid derivative	14.4	326	601	**439**, 353, 335, 179	−	+
13	Tricaffeoyl hexaric acid (II)	14.8	328	695	**533, 371**, 209	−	+
14	Tricaffeoyl hexaric acid (III)	15.0	328	695	**533, 371**, 209	−	+
15	Hydroxybutyryl-tricaffeoyl hexaric acid (leontopodic acid)	16.1	327	781	**619**, 457, 295	+	−
16	Tetracaffeoyl hexaric acid	19.5	328	857	**698**, 533, 371, 209	+	−
17	Isobutyryl-dicaffeoyl quinic acid	19.8	328	585	497, **423**, 335, 179	−	+
18	Methylbutyryl/isovaleryl-dicaffeoyl quinic acid	23.7	328	599	497, **437**, 335, 179	−	+
19	Isobutyryl-tricaffeoyl hexaric acid	24.6	328	765	**603**, 441, 279	+	−

^1^ MS^2^ ions in bold—most abundant ion peak; ^2^ roots; ^3^ aerial parts.

**Table 2:** Retention times (t_R_) and contents of major caffeic acid derivatives in leaves of *Xerolekia speciosissima*, *Buphthalmum salicifolium* and *Telekia speciosa*.

Compound	t_R_ [min]	Content in Leaves [% Dry Weight] ^1^		
		*X. speciosissima*	*B. salicifolium*	*T. speciosa*
3-CQA (1)	5.2	0.029 ± 0.010	0.165 ± 0.097	0.016 ± 0.004
5-CQA (2)	7.0	0.654 ± 0.236	0.815 ± 0.330	1.063 ± 0.081
4-CQA (3)	8.0	0.130 ± 0.046	0.033 ± 0.012	0.032 ± 0.003
Dicaffeoylhexaric acid (4)	8.8	0.486 ± 0.142	-	-
3,4-DCQA (8)	22.6	0.718 ± 0.242	0.909 ± 0.262	0.561 ± 0.146
1,5-DCQA (9)	23.4	1.286 ± 0.342	0.536 ± 0.194	0.350 ± 0.085
3,5-DCQA	24.9	-	-	0.232 ± 0.032
4,5-DCQA (11)	31.2	0.029 ± 0.007	0.060 ± 0.010	0.044 ± 0.017
Dicaffeoylquinic acid derivative (12)	36.6	0.020 ± 0.004	-	-
Tricaffeoylhexaric acid (II) (13)	39.2	0.403 ± 0.230	-	-
Isobutyryl-dicaffeoylquinic acid (17)	55.3	0.258 ± 0.101	0.724 ± 0.380	-
Methylbutyryl/isovaleryl-dicaffeoylquinic acid (18)	58.6	0.097 ± 0.043	0.037 ± 0.013	-

^1^ Data are means of four independent measurements (different individual plants) ± SD.

## Abbreviations

^1^H-NMR—proton nuclear magnetic resonance; ADP—adenosine diphosphate; CC—conventional column chromatography; CCL2 (MCP1)—monocyte chemoattractant protein 1; CDCl_3_—deuterated chloroform; CH_3_COOH—acetic acid; CHCl_3_—chloroform; CQA—caffeoylquinic acid; DCQA—dicaffeoylquinic acid; DMSO—dimethyl sulfoxide; ELISA—enzyme-linked immunosorbent assay; EtOAc—ethyl acetate; FACS—fluorescence-activated cell sorting; FBS—fetal bovine serum; f-MLP—*N*-formylmethionine-leucyl-phenylalanine; GAE—gallic acid equivalent; HBSS—Hanks’ balanced salt solution; HCOOH—formic acid; HEPES—4-(2-hydroxyethyl)-1-piperazineethanesulfonic acid; HPLC–DAD—high-performance liquid chromatography with photodiode array detection; IL-1β—interleukin 1 beta; IL-8 (CXCL8)—interleukin 8 (neutrophil chemotactic factor); IUPAC—International Union of Pure and Applied Chemistry; LPS—lipopolysaccharide; MDM2—mouse double minute 2 homolog; MeCN—acetonitrile; MeOD—deuterated methanol; MeOH—methanol; p53—cellular tumor antigen p53; PAF—platelet activating factor; PBS—phosphate-buffered saline; PI—propidium iodide; PMNs—human polymorphonuclear leukocytes; ROS—reactive oxygen species; RPMI—Roswell Park Memorial Institute; SD—standard deviation; TLC—thin layer chromatography; TNF-α—tumor necrosis factor alpha; TPC—total phenolic content; UHPLC-DAD-MS^n^—ultra-performance liquid chromatography with photodiode array and sequential mass spectrometry detection; UV-Vis—ultraviolet-visible spectroscopy.
